# Concept analysis of collaboration in implementing problem-based learning in nursing education

**DOI:** 10.4102/curationis.v39i1.1586

**Published:** 2016-07-28

**Authors:** Mahlasela A. Rakhudu, Mashudu Davhana-Maselesele, Ushanatefe Useh

**Affiliations:** 1 Department of Nursing Sciences, North West University Mafikeng Campus, South Africa

## Abstract

**Objectives:**

The purpose of this concept analysis was to better understand and define collaboration as it relates to the implementation of problem-based learning (PBL).

**Methods:**

The process of concept analysis was conducted in three phases; namely, theoretical or literature review, empirical or fieldwork; and analysis phases. Rodgers’ evolutionary approach was used to clarify the attributes, antecedents, surrogate, related terms and consequences of collaboration in implementing PBL. The search key terms were ‘collaboration’, ‘problem-based learning’, ‘nursing’ and ‘nursing education’. The search was performed in the Cumulative Index to Nursing and Allied Health Literature (CINAHL), Medline and PsycINFO databases. The articles were reviewed for trends that would reflect the current knowledge for collaboration as a concept. Descriptive qualitative study was used to collect data purposively from participants of three universities offering PBL in Republic of South Africa and three hospitals where PBL students are placed

**Results:**

Collaboration in implementing PBL is described as using the following terms: interpersonal, interactive and personal process, shared goal and governance. The antecedents of collaboration include commitment and support; common goal; formal agreement; training and development; and monitoring and evaluation of tools and mechanisms. Consequences of collaboration in implementing PBL are as follows: information, resource and expertise sharing; personal development and mentoring; creation of supportive and nurturing environment; professional socialisation; improved students’ outcomes; and effective utilisation of resources.

**Conclusion:**

Effective collaboration within nursing education and with other healthcare professionals to achieve quality outcomes in an increasingly interdependent higher education system continues to grow in importance.

## Introduction

Collaboration is a crucial component of all aspects of the academic and clinical learning, as it is through this on-going and continuous process that a common vision and common goals and realities are developed and maintained. According to Bankston and Glazer (2013), collaboration and partnerships can have a positive impact on key dimensions of organisational performance, yet the ability to collaborate consistently continues to elude healthcare settings. This lack of collaboration remains a significant challenge for healthcare executives, college deans, practicing nurses and other healthcare professionals (Bankston & Glazer 2013). Given this scenario and the tension between academic and clinical nurses, it becomes imperative to analyse the concept collaboration in the implementation of problem-based learning (PBL) within the nursing education context.

Collaboration is basic to academic enterprise (Daniels & Khanyile 2013; Miller *et al*. 2015). Whether it is coming together to write a paper, evaluations of students or more complex relationships construction around shared research facilities or teaching programmes, most academics will immediately recognise collaboration as endemic to the academy. According to Zamanzadeh *et al*. (2014), collaboration is an essential element of work relationships in any profession, as it is through this on-going and continuous process that a common vision and common goals and realities are developed and maintained.

The purpose of this study was to explore the concept collaboration in implementing PBL. The term collaboration needs to be analysed for effective utilisation and maximisation of its outcomes in nursing education. D’Amour *et al*. (2008) and Montiel-Overall (2005) remind us that collaboration and partnership are much touted values in organisational life today. Collaborating with other key role players, nurses in academia and practice can directly impact nursing education. Collaboration is often useful in effecting changes and achieving outcomes that are more extensive and powerful that could not be achieved by working alone. Kirschling and Erickson (2010) and D’Amour *et al*. (2008) reported collaboration as central in any collective undertaking. This reinforces the need to analyse the concept for effective use in the implementation of PBL.

American Association of Colleges of Nursing (AACN) (2012) and Herrin *et al*. (2006) documented that academia practice are increasingly viewed as requisite for the future of nursing and paramount to bridging the education preparation and achievement of excellence in professional practice. Collaboration in implementing PBL may assist in bridging the gap between academia and nursing practice. Thus, collaboration is a necessary requisite for assuring a qualified workforce for future and positioning nurses to address emerging healthcare needs (Kirschling & Erickson 2010). Therefore, in attempt to improve collaboration in implementing PBL, it is necessary to conceptualise the term collaboration for effective implementation of PBL in nursing education.

Several publications describe a variety of collaborative models: inter-professional (Bridges *et al*. 2015; Lumague *et al*. 2008; Sullivan *et al*. 2015; Zamanzadeh *et al*. 2014) and collaborative learning (Furber *et al*. 2004), yet, few have explored inter-institutional and intra-institutional collaboration in implementing PBL in nursing, especially in the South African context. The inter-professional collaboration in implementing PBL resulted from the healthcare system that requires provision of collaborative and seamless services (Furber *et al*. 2004). This necessitated this concept analysis to better understand and define collaboration as it relates to the implementation of PBL.

Other models of collaboration in nursing education exist among colleges and universities in implementing collaborative partnerships in Ontario, Canada, with the aim to design, disseminate and evaluate a faculty development programme in nursing (Matthew-Maich *et al*. 2007) and an amalgamation of colleges for the implementation of PBL (Drummond-Young *et al*. 2010). Dearth of literature on collaboration in implementing PBL nursing education in South African context has been found. For this reason, conceptualisation of the term collaboration for effective implementation of PBL in nursing education is very critical.

### Problem-based learning in nursing education

There is increasing pressure on nursing education institutions (NEIs) to focus on developing clinicians prepared to work in rapidly changing, multicultural environments influenced by technological advances and fiscal constraints and burden of diseases (Khatiban & Sangestani 2014). These conditions mandate NEIs to adopt participatory pedagogies that enhance critical decision making, problem solving and team work. PBL is regarded as the most innovative instructional strategy used in health sciences education. Its effectiveness in problem solving, self-directed learning and collaborative skills has been reported widely in higher education (Albanese 2000; Barrows 2003; Billings & Halstead 2010; Hung 2009; Koh, Khoo & Wong 2008; Rideout 2001).

Boud and Felettie (2001) describe PBL as both a curriculum and a practice to provide stimulus for learning. These problems are used to engage students’ curiosity and initiate learning of subject matter (Koh *et al*. 2008; Savery 2006). According to Carlisle and Ibbotson (2005), PBL can also be used as a framework for programmes, curricula, modules and courses.

Given the benefits of PBL, the School of Nursing Sciences at North West University (NWU) adopted PBL for Pre-registration nursing education in 2002, which was evaluated in 2008 from the students’ perspective and by External Programme Evaluation (EPE). Collaboration between clinical and academia practice in implementing PBL was recommended by both students and panellists of EPE for effective implementation of this valuable learning teaching modality. In an attempt to improve collaboration in implementing PBL, it is necessary to conceptualise the term collaboration for effective implementation of PBL in nursing education.

## Purpose of the study

The purpose of this concept analysis was to better understand and define collaboration as it relates to the implementation of PBL.

## Methodology

The process of concept analysis was conducted in three phases; namely, theoretical or literature review; empirical or fieldwork; and analysis phases.

### Theoretical phase: literature review

Rogers’ evolutionary method was used for this literature-based concept analysis (Rodgers & Knalf 2000). In this study, the researcher reviewed the literature to identify data relevant to the attributes of collaboration in implementing PBL in nursing and its contextual features (antecedents, consequences, surrogate and related terms and referents).

A literature search between the years of 2000 and 2015 in the databases of the Cumulative Index to Nursing and Allied Health Literature (CINAHL), Medline and PsycINFO was conducted. The search key terms were ‘collaboration’, ‘problem-based learning’, ‘nursing’ and ‘nursing education’. Initially, 490 full-text articles and 360 abstracts were retrieved. However, many of the retrieved documents were the same articles indexed in multiple databases. After removing those repetitive documents, 275 articles remained in the study database. Of the retrieved articles, the title and abstract of only those articles that had defined or analysed for the concept of collaboration were selected. Articles from management sciences (*n* = 9) and from education (*n* = 3) were included to give perceptions of collaboration from other sciences. Finally, 36 articles from nursing and allied health sciences were included in the final analysis of the concept of collaboration. Dictionaries were also used to obtain a general definition of collaboration.

### Empirical phase: Fieldwork

This stage of the study involved an exploratory descriptive qualitative study. The fieldwork phase was conducted with an aim of exploring the concept using empirical data. The aim of this phase was to refine the findings of the theoretical phase. Semi-structured interviews were used to collect data from purposively selected participants (Creswell 2014; Munhall 2012) to enrich and contextualise the concept. The following criteria were used to select participants who would be best to answer the interview question (Creswell 2014; Munhall 2012): (1) Nurse educators: Educator registered with South African Nursing Council and teaching at a university; possessing at least 2 years teaching experience at a tertiary nursing education institution and implementing PBL to ensure adequate exposure to PBL and employed in an institution that offers PBL ensuring equality of participants. (2) Nurse Managers: the inclusion criteria for the managers are from health settings where students are placed for clinical learning, have at least 2 years’ experience and above in management position. (3) Universities: The university should be offering PBL in pre-registration nursing programme; (4) Clinical Facility: clinical facility is where PBL nursing students are placed for clinical learning in the North-West Province.

*Data collection*: Interviews lasted for 30–60 minutes. The interview guide consisted of questions such as ‘Can you tell me what you understand about the concept of collaboration?’, ‘How should collaboration in implementing PBL be?’ ‘What could be the consequences or results of collaboration in implementing PBL?’ Interviews were audio taped, and the researcher made additional notes on non-verbal, para-verbal and verbal observations (Munhall 2012).

*Ethical measures*: Volunteers who fitted the criteria were recruited to participate and requested to give written consent after expectations of their participation were explained. Participation was voluntary. Ethical clearance was obtained from the NWU (Ethics number: NWU-00033-11-A9). Permission from the North-West provincial Department of Health, and authorities of participating hospitals and universities was obtained. The identities of the institutions and individuals were maintained confidential by the use of codes, rather than names.

*Quality measures*: In qualitative research, the researcher works with truths that are socially situated. Thus, measures for ensuring trustworthiness of the findings by Guba (in Krefting 1991) were utilised. The criteria that typify data verification in this study are summarised in Table 1.

**TABLE 1:** Strategies to ensure truth value.

Criterion	Description
Member checking	The findings was taken back to participants and ensuring that they agree (Green & Thouragood 2009).
Reference adequacy	The transcribed interviews, the research protocol and re-order’s protocol were in the final report, in order to increase reference adequacy.
Authority of the researcher	The researcher has experience of qualitative research in Master’s studies and was guided by a high-rated researcher who is acting as a promoter.

*Source*: Green and Thouragood 2009 and Streubert-Speziale and Carpenter 2007

### Analysis phase

Consistent with the evolutionary approach to Concept Analysis (Rodgers & Knalf 2000), an inductive process was implemented to identify relevant aspects of collaboration. An inductive approach was used to analyse the empirical data from the opinions of nurses regarding collaboration in implementing PBL in nursing education (Rodgers & Knalf 2000). In addition, the following reasoning strategies were used for concept analysis in this study: analysis, synthesis, deduction, inferences and derivation as suggested by Chinn and Krammer (2011).

## Results of literature review

### Identification of the concept of interest

In order to define and describe the concept ‘collaboration’ in implementing PBL to its attributes, the word is defined in a general sense from the dictionary, after which it was defined using subject-specific literature to place it in the proper context.

### General definition of concept collaboration

Collaboration is an intricate concept with multiple attributes. It is defined in a variety of ways, many of them explicitly referring to interdisciplinary collaboration. Collaboration may be defined as ‘the act of working jointly’ (Collins Dictionary 2009). Webster Comprehensive Dictionary edited by Marckwardt, Cassidy and McMillan (2009) describe collaboration as 1) to labor or cooperate with another, especially in literary or scientific pursuit; 2) to cooperate willingly and traitorously with an enemy. In this study, it refers to working jointly in implementing PBL in nursing education.

### Subject-related definitions of concept ‘collaboration’

In business dictionary by Collin (2006), collaboration is described as an agreement between two or more partners to share knowledge or resources which could be beneficial. It is an act of working together on a project.

In business management, collaboration can be found both inter- and intra-organisationally (Eisingerich & Bell 2008) and ranges from the simplicity of a partnership and crowd funding to the complexity of a multinational corporation. Collaboration between team members allows better communication within an organisation and throughout the supply chains. It is a way of coordinating different ideas from numerous people to generate a wide variety of knowledge (Eisinrich, Rubera & Seifert 2009).

Dorner, Taylor and Hodson-Carlton (2001) defined collaboration as a process of shared creation. Individuals with complementary skills interact to ‘create a shared understanding that none had previously possessed or could have come to on their own’. These authors also indicate that collaboration requires that all parties are working towards a common goal. In this case, the goal of collaboration is effective implementation of PBL in nursing education.

Boughzala and De Vreede (2015) defined collaboration in management as a process of exchanging information, sharing and enhancing capacity for mutual benefits and common goals. In management, a significant number of organisations use collaborative work practices to help achieve success.

Collaboration is identified within the supply chain management discipline as a strategy that helps to link inter-institutional business research operations in order to achieve a shared market opportunity (Cao & Zhang 2011). Through collaboration, institutions should aim at maintaining a competitive advantage in their core areas of operation.

In human resource management, collaboration is defined by Bedwell *et al*. (2012) as ‘an evolving process where two or more social entities actively and reciprocally engage in joint activities aimed at achieving at least one shared goal’. The definition gives the most critical attributes of collaboration, namely, process, two or more entities, active and reciprocal engagement and shared goals.

Collaboration in education–two or more equal individuals voluntarily bring their knowledge and experience together by interacting towards a common goal in the best interest of students for the betterment of their educational success (Mfum-Mensah 2011).

### Identification of defining attributes

According to Rodgers and Knalf (2000), attributes of the concept constitute a real definition, as opposed to nominal or dictionary definitions that merely substitute one synonym expression.

In reviewing the literature, it was noted that a formal definition of collaboration in implementing PBL in nursing education was not provided. Instead, collaboration was placed on nursing education, practice, clinical learning and research. However, various attributes of collaboration in healthcare practice and education were consistently noted in literature from nursing, medicine, management and business and education.

The critical underlying assumptions regarding collaborations drawn from literature are discussed hereunder.

### Collaboration is an evolving process

The literature has conceptualised collaboration as a process (Bedwell *et al*. 2012; Patel, Pettitt & Wilson 2012). By conceptualising collaboration as a process that involves parties interacting together, this definition retains the dynamic and evolving nature pervasive in definitions across disciplines (D’Amour *et al*. 2005). Collaboration is not static but dynamic and changes over time. Furthermore, collaboration is an active process involving interpersonal interactions and relationships that change over time (Bedwell *et al*. 2012; Patel *et al*. 2012). Collaboration is viewed as a process that can evolve, improve and change over the course of its life cycle.

### Collaboration is transformational

Collaboration is transforming in the sense that you do not leave the same way you came in: (1) there’s some sort of change; (2) you give up part of yourself; (3) something new has to be created and (4) something happens differently because of the process (Thomson & Perry 2006).

### Collaboration requires two or more social entities

A significant attribute of collaboration is that two or more individuals must be involved in a joint venture, typically one of an intellectual nature. Collaboration is seen by scholars in organisational behaviour, sociology and anthropology as a process that involves interaction among social units, including people and organisations (Longoria 2005). For both social interaction and working together, two or more entities are required. Moreover, collaboration can occur between a variety of entities, including ‘individuals, groups, organisations, or even societies’ (Longoria 2005). The term entity is used to refer to individuals, teams, units, departments, functional areas and organisations (Bedwell *et al*. 2012). Collaboration is not limited to just the same level of entities (i.e. two organisations or two teams), but rather it can also occur across levels. Collaboration can occur (1) between individuals and organisations and (2) across levels of analysis and involve any combination of individuals and organisations.

### Collaboration is reciprocal

Henneman, Lee and Cohen (1995) have described collaboration as distinctly reciprocal. Collaboration cannot be one-sided; rather, it requires active, mutual engagement in the collaborative process at some level from all involved parties (Longoria 2005). More simply stated, one party dictating and controlling another party cannot be considered collaboration as this type of interaction would be better defined as delegation of work, or even as coercion. It is critical that all involved entities work interdependently and contribute sufficiently towards reaching their joint aim (Bedwell *et al*. 2012). In essence, collaboration is a back-and-forth reciprocal process that requires each involved party to actively contribute in some way across the lifecycle of collaborative effort.

### Collaboration requires sharing

The attribute of sharing is used liberally in the literature to describe characteristics (AACN 2012; D’Amour *et al*. 2005; Dorner *et al*. 2001; Patel *et al*. 2012). Most notably, attribute is sharing goal objectives, responsibility, decision making and power. The presence of shared goals, objectives or vision is necessary to ensure that collaborators are united and working towards a common outcome.

Sharing of power, resources and expertise are important in the quest for improving the quality of nursing care (AACN 2012). Shared planning and decision making, a team approach, shared responsibility and shared power are all requisites of collaboration in clinical learning. As an attribute for collaboration in implementing PBL in nursing education, sharing implies equal participation of all collaborators.

Sharing of responsibility implies that involved partners cooperate in the process of collaboration and have accountability (D’Amour *et al*. 2005; Henneman *et al*. 1995). Shared decision making ensures that the perspectives of all collaborators are taken into consideration during the planning and implementation of PBL in nursing education. Collaborative partners in implementing PBL need sharing of power, resources and expertise in the quest of improving nursing education. Shared planning and decision making, team approach, shared responsibility and shared power are all characteristics of collaboration (Bedwell *et al*. 2012).

### Collaboration requires communication

Communication in collaborative work underpins how people understand each other and how knowledge is transferred (Patel *et al*. 2012; Horwath & Morrison 2007). Communication should be open to enable informal and formal exchange of tasks and contextual information to support collaboration (Cheng *et al*. 2016; Dorner *et al*. 2001). The overriding ingredient for collaboration in implementing PBL in nursing education is communication, communication and more communication. For example, routine information calls, calls for help, helps for proactive assistance and other similar calls are crucial for success. Open, frequent, balanced, two-way, multilevel communication is generally an indication of close collaboration (Cao & Zhang 2011; Horwarth & Morrison 2007). Establishment of effective communications one-on-one, among subgroups, web-based, telephonic and with printed information (quantitative and qualitative) contributes to a successful collaboration.

### Trust is a must in collaboration

Good collaborative efforts are characterised by mutual trust and respect, and these should be established early in collaboration. According to Patel *et al*. (2012), people are more likely to trust those who are similar to themselves (e.g. in age, status, cultural, professional and educational background). Trust, willingness to communicate and sharing information openly indicate individual and organisational readiness to collaborate in any endeavour including implementing PBL (Cheng *et al*. 2016; Daniels & Khanyile 2013; Patel *et al*. 2012).

### Equality in relation

AACN (2012) viewed equality among the collaborators as an indicator of collaboration. Collaborative efforts should focus on becoming peers. Collaboration requires partners to see themselves as peers and to share a sense of ownership. Carnwell and Carson (n.d.) refer to this as non-hierarchical relationship.

These views highlight the common attributes that emerged from the literature review on collaboration. Defining attributes that emerged in the literature in relation with collaboration include (1) commitment; (2) intellectual and cooperative endeavour; (3) joint venture; (4) participation in planning and decision making; (5) equality or non-hierarchical relationships; (6) willingness to work together towards agreed purpose and (7) trust and respect for collaborators (Barnett *et al*. 2009; Henneman *et al*. 1995; Kinnaman & Bleich 2004; Mfum-Mensah 2011).

### Identification of surrogate terms

Rodgers and Knalf (2000) describe surrogate terms as means of expressing the concept other than the word or expression selected by the researcher, meaning that the concept can be expressed in many ways. Collaboration is frequently equated with (1) an alliance, (2) association or (3) partnership, characterised by mutual goals and commitments (Henneman *et al*. 1995). This section will describe the surrogate terms for collaboration in implementing PBL.

*Association*: Collins’ Dictionary (2009) defines an association as a group of people with a common interest. In this context, collaboration in implementation of PBL is viewed as a group of collaborative partners with a common interest.

*Alliance*: This refers to an association of two or more people for a common goal (Collins’ Dictionary (2009).

*Partnership*: This is defined by Collins’ Dictionary (2009) as a relationship in which two or more people or organisations work together in business venture. Casey (2011) is of the opinion that successful partnerships are non-hierarchical and partners share decision making and common ownership of the resolution of challenges. There is agreement in literature that collaboration is a relationship that involves commitment to improvement of performance, efficiency and consideration of partners’ rights in the context of major decisions (Casey 2011; Harvath *et al*. 2007).

### Identification of antecedents

Rodgers and Knalf (2000) suggest that concepts have antecedents, which are events or circumstances that happen or exist prior to the concept occurs. In literature review, more antecedents than attributes were offered for concept collaboration. It was also noted that several terms cited as antecedents for collaboration were noted as attributes by others. Below are antecedents that emerged from analysis of collaboration as a concept in implementing PBL.

#### Commitment and support

The critical antecedent condition is recognition of the importance of individual and organisational commitment to ensure that collaboration is a success (Barnett *et al*. 2010; Daniels & Khanyile 2013; Patel *et al*. 2012). Elements necessary for collaboration to succeed are commitment of time, energy and resources. Leadership and commitment are basic to the success of any collaborative endeavour including the implementation of PBL in nursing education. Clear and unequivocal support from institutional authorities and uniform alignment to achievement of a common goal are critical for success of collaboration in implementing PBL.

Two levels of support and commitment are discussed as important for collaboration to be successful: organisational or managerial commitment and support are recognised in the form of education, resources and rewards (Petri 2010). In individual commitment and support, each individual must have the desire to participate or believe in the interdisciplinary collaboration (Petri 2010). With commitment, support and encouragement from management levels of collaborating organisation, success in collaboration is possible (Barnett *et al*. 2010).

#### Common goal

A shared mission and common goal should guide the collaboration. Patel *et al*. (2012) state that a clear, common vision and objectives can provide a framework within which collaboration strategies and goals determine the success of collaborative project. Good collaboration requires participants to have a clear understanding of tasks and collaboration goals and objectives. For collaboration in implementing PBL, a well-defined common goal will provide a common ground and understanding of tasks, roles and responsibilities of collaboration.

#### Formal agreement

The other antecedent condition is a formal written agreement describing the type and level of collaboration and various roles as well as the responsibilities of collaborators (D’Amour *et al*. 2008; Dorner 2001). A memorandum of understanding (MOU) should be in place stating the type and level of collaboration, roles and responsibilities of each collaborator, time frame of collaboration as well as the process to be followed to achieve the goal. Collaboration is aided when an individual’s and an organisation’s roles are coordinated. Individual and inter-organisational collaboration require particular effort for participating members to have understanding of roles and responsibilities in the different organisations (Patel *et al*. 2012). Therefore, a formal agreement is mandatory for a successful collaboration in implementing PBL in nursing education.

#### Active participation

Active participation in decision making, design and implementation of collaborative venture is basic to collaboration in implementing PBL. The more people participate in these processes, the more likely each will feel ownership and therefore on-going commitment to the collaboration (Mfum-Mensah 2011). Active participation of partners in the formal, structured, collaboration is a must to ensure that each contribute to shared vision (Carnwell & Carson n.d.; Hendrix *et al*. 2011)

#### Training and development

Training and development are required for task completion, collaboration tools and collaboration itself (Daniels & Khanyile 2013; Patel *et al*. 2012). These authors are of the opinion that personal training and development are associated with improved productivity and collaborator satisfaction. Training and development of collaborators for implementation of PBL provide opportunities for the team members to acquire new skills or improve the existing skills and develop shared mental models, and this can improve institutional performance. Therefore, it appears that for the success of this collaboration, institutions should be aware of skills and behaviours required to perform particular collaborative tasks or functions and base.

#### Monitoring and evaluation

Monitoring and evaluation of collaboration are required to assess the success and take corrective measures where flaws are identified. Evaluating collaboration effectiveness involves assessing how the partners work together to achieve outcomes and whether the collaborators are able to work together in future (Patel *et al*. 2012). For collaboration in implementing PBL, monitoring and evaluation involve assessing individuals as well as collective efforts, depending on the type of task and responsibility as both can have important influence of the overall performance of collaboration. Commitment to on-going evaluation of evidence-based performance improvement is a requirement for effective collaboration.

In summary, antecedents to collaboration can be classified under personnel and organisational or environmental factors.

*Personnel factors* include the following:

- Sufficient educational preparation;
- Clear understanding and acceptance of their role and expertise;
- Effective communication;
- Respect for and understanding of other’s role;
- Sharing knowledge, values; responsibility, vision and outcomes; and
- Talent, tact and trust have been identified as essential ingredients for effective collaborative partnerships (Carnwell & Carson n.d.; Hendrix *et al*. 2011).

*Organisational/environmental factors*: Environmental factors include the elements that lie outside the collaborators. The first antecedent condition in this category is recognition of the importance of organisational commitment to ensure that the loss of individuals would not affect the viability of the partnership. Other factors include the following:

- Shared mission and goal;
- Equality or non-hierarchical relationships wherein collaborators can act autonomously;
- Formalisation of agreements; and
- Allocation of time as a resource (Carnwell & Carson n.d.; Hendrix *et al*. 2011).

### Identification of related concepts

Coordination, cooperation, teamwork and collaboration: more often than not, these words are used interchangeably (Bedwell *et al*. 2012).

*Teamwork*: Teamwork is multidimensional and represents processes that involve two or more entities actively and reciprocally working towards achievement of a shared goal (Bedwell *et al*. 2012; Cheng *et al*. 2016). Therefore, it is necessary for collaborators in implementing PBL in nursing education to have teamwork.

*Coordination*: Bedwell *et al*. (2012) describe coordination as another concept frequently used to describe collaboration (often at the team level). This concept refers to the sequencing of interdependencies to most efficiently accomplish work tasks (Marks, Mathieu & Zaccaro 2001). Similar to collaboration, coordination can involve two or more social entities; however, it can also describe two or more resources that are non-social in nature. This means that collaborative partners need coordination in implementing PBL in nursing education.

*Cooperation*: Cooperation is another concept used interchangeably with collaboration. It is defined as a process of working or acting together or willingly to assist. Cooperation means working together with others. Bedwell *et al*. (2012) view cooperation as an attitudinal concept describing the extent to which entities are concerned about the overall goal rather than individual goal, thus helping to facilitate the process of collaboration. In collaboration in implementing PBL in nursing education, cooperation is necessary for success.

## Results of empirical phase

Individual semi-structured interviews (*n* = 11) and five focus group discussions (*n* = 33) were conducted from June 2011 to May 2012 from purposive recruited participants from three out of five universities offering PBL in South Africa and three hospitals where PBL nursing students are placed in the North West province. Realisation of the qualitative sample was achieved after interviewing 11 (*n* = 11) participants and 5 focus group discussions (FGD) (*n* = 33). Ages of participants varied from 31 years to 64 years, and the highest qualification of participants was a PhD in nursing and the lowest, a post-basic Nursing Diploma in various clinical specialisations. The majority (*n* = 17) of nurse educators had master’s degrees in nursing education. Most (*n* = 38) were women, while only six (*n* = 6) were men.

### Defining collaboration in implementing problem-based learning

Collaboration was defined by a majority of participants as the process of working together by both partners towards achieving the same goal. This is what one manager said:

‘Collaboration in implementing PBL is a process wherein nursing education institutions and clinical staff work jointly to educate the student through PBL. We need to work collaborative using collegial relations to plan the PBL curriculum up to evaluation of students. Agreements or MOU must be in place with executive managerial commitment to collaboration.’

### Antecedents of collaboration

Antecedents of collaboration included (1) managerial commitment, (2) common goal, (3) capacity building on PBL, (4) communication, (5) contractual agreement and (6) mutual trust and respect.

#### Managerial commitment

The participants perceived commitment of strategic managers as necessary for the success of the collaboration in implementing PBL. The following are the quotes from the participants:

‘The nurse mangers, especially at strategic position should be involved from the planning phase of problem-based learning curriculum.’ (Participant 6)

Another had this to say:

‘Like I said earlier, our top management should know about this collaboration. If possible … they should also be trained on PBL. They should inform about PBL so that they know and be able to support at operational level.’ (Participant 12)

#### Common goal

A significant attribute of collaboration is that two or more individuals must be involved in a joint venture, typically one of an intellectual nature with a common goal (Henneman *et al*. 1995; Zamanzadeh *et al*. 2014). The participants perceived having a common goal as leading to successful collaboration in implementing of PBL. These are the statements of participants:

‘Common goal in collaboration is very important in any collaboration. In this instance, our common goal will be training a nurse through PBL implementation. If we have shared mission, vision and goals, our collaboration will be in the right direction.’ (Participant 8)

#### Contract/Agreement

In any collaboration, there must be an agreement about what problem is to be addressed and how multiple problems are to be prioritised (D’Amour *et al*. 2008).

The participants verbalised the need for a contract or a MOU. This requirement was verbalised in different ways. Some referred to this as service level agreement, agency agreement and agreement contract.

One nursing manager said in relation to formalisation of collaboration:

‘Generally, for collaboration to be formalized…. It’s basically for people to come together. Either coming together or engaging new issues of LSA, that is, level of service agreement…. Where they would then understand as we work together, this is what I am expecting from this partner, and this is what I should be giving that particular partner or collaborator.’ (Participant 1)

Another said this:

‘I think we should have a binding agreement. We should sign an agreement that will bind all of us to ensure how this collaboration is going to unfold. In the agreement, we will have terms of reference in terms of meetings, training, assessing students, in terms of joint appointment of preceptors or whatever.’ (Participant 7)

#### Continuous development of all collaborators on problem-based learning

Participants perceived the need to develop the collaborators on problem-based learning (PBL) so that the collaborators are conversant with this teaching strategy. The statements below illustrate the need for training in PBL for success of the collaboration in implementing PBL in nursing education. One participant said this:

‘The university and the college must also train and develop the nurse managers on the use of PBL so that we are able to speak the same language. The clinical staff should be invited to the workshops on PBL.’ (Participant 4)

This is supported by Williams and Beatti (2008) in their systematic review wherein they suggested that clinical personnel need training and development on PBL in order to assist undergraduate health professionals in PBL. Findings by Dornan *et al*. (2007) confirm the need for the development of clinical professionals on PBL as PBL methods did not automatically transfer in clinical teaching.

#### Monitoring and evaluation in collaboration

Monitoring and evaluation of collaboration activities, projects and programmes are increasingly recognised as important functions of every collaborator in healthcare and education settings. In many instances, collaboration in project and activities are hampered because little attention is given to monitoring and evaluation. Participants perceived M&E as vital components for a successful collaboration in implanting PBL. This point was clearly expressed by participants.

One nurse educator verbalised thus:

‘I think we should also develop something like an evaluation tool to evaluate if everybody is following the agreement, and to see if the collaboration is working.’ (Participant 18)

One nurse manager said this:

‘Another critical aspect for a successful collaboration is a continuous monitoring and evaluation to assess the progress and success of the collaborative partnerships in implementing PBL. The collaboration can be assessed regularly by the collaborators including the recipients of the collaboration, namely, the students. Clients satisfaction interviews, self-evaluations using partnerships tools.’ (Participant 6)

Literature (Crosby & Bryson 2010; Owen & Grealish 2006; Patel *et al*. 2012) supports the findings that monitoring and evaluation of collaborative processes to ensure that decisions agreed upon are honoured within the scope and parameters. Mutual performance monitoring is associated with effective teams and involves team members monitoring each other’s work in collaboration (Patel *et al*. 2012).

### Defining attributes of collaboration

Successful collaboration is characterised by clear communication, mutual understanding and respect.

### Communication

Successful collaboration is characterised by clear communication, true dialogue, active listening, an awareness and appreciation of differences and the ability to negotiate options (AACN 2012; Bankston & Glazer 2013; Miller *et al*. 2015; Zamanzadeh *et al*. 2014; Horwath & Morrison 2007). Participants verbalised communication as a critical tool in any collaborative effort. The following is what one of the managers verbalised:

‘Communication is a very important tool in any partnership. There must be open and regular communication between the collaborators. It can be through regular meetings, written communication, for example through memos, e-mails. But schedule meetings with clear agendas will contribute to success.’ (Participant 3)

The AACN (2012) recognised that open communication through a culture of trust and respect fosters partnership success.

### Mutual understanding and respect

Re-requisite to effective collaboration include mutual understanding and collaborators’ tolerance for sharing power and willingness to adapt their operations and procedures to facilitate the partnership’s performance (Bankston & Glazer 2013; Brinkerhoff 2002; Daniels & Khanyile 2013; Miller *et al*. 2015; Montiel-Overall 2005).

Participants verbalised mutual understanding and respect as necessary for collaboration in the implementation of PBL to work. This is a quote from participants:

‘I think mutual understanding and respect are very vital for the collaboration to succeed. If we do not respect each other as nurse managers and educators problems will erupt and students may pick that up and use it as excuses not to do their work.’ (Participant 21)

This concurs with Derbyshire and Machin (2011) who view reciprocal role awareness and mutual respect as important for collaborative partnerships.

### Non-hierarchical relationship

Non-hierarchical relationships, mutual trust and respect are necessary for a successful collaboration in implementing PBL. A non-hierarchical relationship refers to relationships wherein no collaborators are perceived as senior to others. According to the Free Dictionary (www.thefreedictionary.com), non-hierarchical refers to a relationship that is not classified according to successive levels or layers.

Participants perceived non-hierarchical relationships as vital for the success of collaborations. The following are the quotes from participants:

‘I mean, all of us must know that when you are in this collaboration when we are in meeting; we are equal. Nobody is more important than the other.’ (Participant 10)

This perception is in line with Casey (2011), Owen and Grealish (2006), Harvath *et al*. (2007) and Carnwell and Carson (n.d.) who viewed successful collaborative relationships as dependent on team-oriented environment with non-hierarchical structures and where the participants share decision making and common ownership of the resolution of challenges.

Equality or flattened hierarchical relations are likely to be another conscious strategy to nurture and strengthen collaboration in implementing PBL in nursing education.

### Active participation of collaborators

Participants viewed participation and involvement of collaborative partners in planning commencing with goal setting as critical for the success of the project.

This was articulated by one nurse educator:

‘Again, collaboration in implementing PBL requires active participation of nurses from the clinical space. They should be involved from the beginning, for example planning of the PBL curriculum, implementation and evaluation. Clinical practitioners will and can contribute a lot during scenario development, facilitation of clinical learning as well as evaluation of PBL clinical learning.’ (Participant 17)

Patel *et al*. (2012) and Brinkerhoff (2002) emphasise active participation of all member partners according to their cooperative advantage and agreed roles. This includes decision making, as well as participation in meetings and involvement in relevant discussions and programme activities.

### Defining consequences of collaboration

Consequences of collaboration are for staff, students and organisations. The participants are of the opinion that collaboration has consequences in many aspects.

### Consequences for staff

The participants all agreed that collaboration will result in information and expertise sharing, personal development and mentoring.

### Information and expertise sharing

Participants perceived collaboration as beneficial for information and expertise sharing.

The following are quotes from participants:

‘Growth in the sense that … that … if we collaborate, we share information, then this will encourage research on the subject and contribute more, like we are doing now. This will increase the knowledge on the subject PBL.’ (Participant 22)

This is what one nurse manager said:

‘I also think we as managers will be able to learn more about the subject (PBL). We will also learn more about new developments in health care service. I think if we collaborate, we will be able to share information and expertise. It will give us the clinical staff an opportunity to contribute toward education of our future practitioners.’

This is supported by Lehna and Byrne (1995) and Miller *et al*. (2015) who noted the benefits of collaboration to include increased theoretical knowledge and visibility and increased interest, participation and personal growth. They indicated further that educators will develop awareness of clinical requirements in the various settings (AACN 2012; Brinkerhoff 2002).

### Personal development and mentoring

Participants viewed collaboration in implementing PBL as contributing to personal development and growth. The following are quotes from participants:

Quote from the nurse educator

‘In collaboration with champions in PBL like McMaster University we are like to learn from the best and we will be mentored on various aspects of PBL thus effective implementation thereof.’ (Participant 4)

These findings support the results of Williams-Barnard *et al*. (2006), wherein the practice nurses valued collaboration in nursing education in terms of increase in professional and intellectual stimulation, enhancing awareness of learning processes, sharing and building knowledge base for nursing education practice and continuing education.

### Consequences for students

Students as internal customers of nursing education and PBL will benefit from collaboration. Within the professional discipline of nursing, excellence in practice can best be attained when those in education and practice setting combine their efforts and talents in collaboration. The consequences that emerged here include collaboration, professional socialisation and obtaining the best from the expertise of collaborators.

### Professional socialisation

Professional socialisation for nursing students can be an act of balance, finding a way to fit in appropriately, while moving between the diverse and sometimes conflicting values of the colleges of nursing and the healthcare organisations. The participants were of the opinion that collaboration will assist in professional socialisation of the students. This is what one participant said:

‘I also believe that if those people in the clinical areas can be invited to collaborate in planning clinical learning and evaluation of PBL students. The students will greatly benefit from such a partnership. Students will also learn about collaboration as we would be role modelling collaboration to them. They will learn by imitation.’ (Participant 18)

These findings concur with Ardahan, Akcusu and Engin (2010) and Brown (2009). In their study of interdisciplinary collaboration, students expressed more positive attitudes towards collaboration and helped in the development of awareness of team working and roles of each other. Student-centred collaboration can be a functional reality in the delivery of quality education (Brown 2009). These authors also suggest that collaboration can be used as a strategy to enhance active learning across disciplines and prepare students for collaborative interactions they will experience in future employment situations (Ardahan *et al*. 2010; Brown 2009).

### Obtain the best from the expertise of collaborators

Participants indicated that the students will benefit from expertise of the collaborators. For nursing education, students will benefit from expertise of collaborators as illustrated in this comment about this benefit:

‘Specialized nurses from different unit can be invited for scenario development, teaching in class and evaluation of students. In this way, the students will be benefiting from expertise of both clinical and teaching staff. If we collaborate, the clinical staff will be involved in all curricular activities from planning of PBL curriculum, scenario development and evaluations.’ (Participant 30)

This concurs with the study of Connolly and Wilson (2008), who found that students receive real-world clinical instruction from competent and credible clinicians and service guaranteed students for clinical site, fostering recruitment into extern programmes and new graduate into positions.

### Quality nursing education

The statement below describes quality nursing education as a benefit according to one nurse educator:

‘So I think that this improved or build their knowledge and skills … and motivation as well, I think they have started to implement some things that I am seeing I don’t think it only benefit the students of our programme and improves the quality of our students. As collaborators we will be providing a nurturing environment for students promoting quality education.’ (Participant 12)

These findings are backed by Alberto and Hearth (2009), who conclude that collaborative efforts in nursing education have innumerable benefits, such as exposure of students to expert areas that they would otherwise only read about and discuss with the faculty.

The results suggest that collaboration in implementing PBL in nursing education enhances the students’ capabilities. Collaboration is thus helpful in achieving quality outputs and increased productive learning and thus quality education.

### Consequences for the collaborating organisations

Collaboration has the potential to benefit the partnering organisation. The following consequences emerged from this category: (1) Sharing of resources and facilities and (2) effective utilisation of resources.

### Sharing of resources and facilities

Participants expressed that collaboration in implementing PBL in nursing education would promote sharing of resources. The statement below depicts sharing of resources as a positive outcome of collaboration:

‘Um … generally, one would still say … we have limited resources, especially in implementation. Resources like people, time, talents, library, computers and other teaching equipment can be shared for the benefit of the students.’ (Participant 9)

The findings are in line with those from an integrative review by Beal (2012), which reveals that collaborative partnerships enhance sharing of resources and facilities. According to Bleich *et al*. (2004), who are regarded as thought leaders on topic of academic service collaborative partnerships in nursing, the benefits that are cited by AACN in 1990 remain today. These include maximisation of resources and sharing. The findings are supported by literature on inter-institutional collaboration. Fisher, Kathryn and Peek (2009) viewed inter-institutional network as a possible strategy to draw on and leverage the existing resources of collaborating organisations. It appears that collaboration in implementing PBL in nursing education has the potential for enhancing organisational sharing of resources with collaborative partners.

### Effective utilisation of resources

Participants viewed collaboration as beneficial as it promotes effective utilisation of minimal or scared resources. As one nurse manager interviewed puts it:

‘I think as collaborators we will be able to share information and resources, both at individual and institutional level as well as team level. Institutions will share the limited resources and thus promoting effective utilization of the resources.’ (Participant 14)

Bankston and Glazer (2013) endorse the view that collaborative and interdisciplinary teamwork is a way to overcome limited financial and personnel resources and to ensure that programmes and knowledge development are responsive to consumer needs.

### Analysis phase

The essential attributes of collaboration were organised and reduced by developing categories and subcategories that would be used to define collaboration in implementing PBL in nursing education.

The purpose of reducing the attributes to essential and related criteria is to ensure that all important criteria are included in the final definition of the concept collaboration. These criteria were then used as the basis for the model case and form the final conceptual definition. Table 2 depicts the essential attributes of collaboration reduced according to categories and subcategories.

**TABLE 2:** The essential attribute from empirical data and those emanating from literature.

Category	Sub-category	Attributes
Antecedents	Personnel	Trust and respect; Skills and positive attitude; Personal commitment and cooperation; and Willingness to work together towards agreed purpose.
	Organisational Environment	Managerial commitment and support; Shared governance and decision making, shared goal and objectives; Formal agreement contract; Equality or non-hierarchical relations.
	Planning	Shared planning and decision making; Training and development of collaborators; and Identification of resources needed for collaboration.
Attributes	Process	Interpersonal process Two or more entities Training of collaborators Requires monitoring and evaluation Communication
Consequences	Collaborators	Development and growth; Information and expertise sharing; Mentoring
	Students	Supportive and nurturing environment; Quality education; Professional socialisation; and Improved student’s outcomes.
	Organisation	Improved productivity and effective use of personnel; Inter-professional cohesiveness.

*Source*: Authors own work

### Model case(s)

Ms RG, an executive manager of a nursing education institution, who holds a master’s degree, viewed collaboration in implementing PBL between academia and clinical practice as long overdue. This is what she verbalised:

‘Training of nursing students in any form requires collaboration. Collaborators should include nursing education institution, facilities where PBL students are placed for clinical learning and centres of excellence in PBL as well as with other disciplines within the university such biological and social sciences. Both interdisciplinary and inter-institutional collaborations are important for effective implementation of PBL. I think for collaboration in implementing PBL to be successful, we need to have a formal agreement that spells out the roles and responsibilities of collaborators. In this collaboration, mutual respect, collegial relations that are non-hierarchical, and adequate and free flowing communication are very critical. No one should be superior to another from all the collaborators. A common goal is critical in collaboration as well as joint planning of PBL curriculum from design and implementation to assessments. It is also important to monitor and evaluate this collaboration. My opinion is that with collaboration, information will be shared, even decision making. People will decide together, also eh‥ eh… eh…. They will also copy something that is good from others. We will share resources, talents and expertise for the benefit of the students who are our clients.’

Mr. TT, a professional nurse manager in the clinical area with more than 15 years in the clinical service verbalised the following:

‘In my opinion, collaboration in implementing PBL, we need to have a common vision and goals. We will benefit greatly from working together and stop blaming each other as clinical and academic personnel especially when students are not doing well. We will share decision making and problem solving. We will also be role modelling collaboration to the students as professional socialization. Our students will be nurtured by experts from academia and clinical services, thus positive outcomes and improved quality education. The gaps between academia will be narrowed if not closed. As professional nurses from the services we will be able to contribute to the education of student and this will add value to nursing education. I view collaboration in this case as a process of joint planning characterized by healthy interpersonal relationships, active participation, cooperation, and commitment from the all the collaborators in nursing education including the multidisciplinary team member. Managerial buying in is critical. I mean commitment from top management is needed and willingness to allocate resources for collaboration.’

### Theoretical definition

Based on this analysis and clarification of the concept of collaboration as well as correlation of theoretical and empirical meanings of collaboration, the following definition is offered in the context of nursing education: collaboration in implementing PBL is a dynamic, interpersonal, interactive, developmental and beneficial process whereby individuals or organisations work jointly to achieve a shared goal through shared governance, decision making and power for a specific period.

## Discussions

Although collaboration is frequently used in different practices including healthcare practice and education, it is more dynamic and complex than the current uses dictate. Concept analysis revealed that collaboration is described differently in different settings, but it is articulated as an interactive, beneficial and developmental process. From the analysis, collaboration is goal oriented wherein talents, energies, information and resources are shared in a non-hierarchical platform.

One notable application of this analysis is that the positive consequences found in literature can be categorised as personal benefits (staff benefits such as information and expertise sharing, personal and professional development and mentoring; and students’ benefits such as professional socialisation, obtaining the best from experience of collaborators, quality education and competent graduate) and organisational benefits such as resource sharing and effective utilisation of resources.

It is also noted that collaboration is used interchangeably with partnership, teamwork and cooperation and could easily serve at different organisational levels; for example, intra-professionally within the nursing profession (midwifery, community health and mental health); interdisciplinary (among disciplines servicing nursing education such as biological science and social sciences) and inter-institutionally (academia and clinical services).

## Conclusion

Collaboration in implementing PBL in nursing education can be reduced to a goal oriented process rather than a structure or outcome. It is a particular process used to achieve a specific outcome, namely effective implementation of PBL. The spin-off benefits of collaboration in implementing PBL are creation of supportive and nurturing environment; inter-professional cohesiveness; improved student’s outcomes; demystifying of education with the bridging of gaps between fragmented services and sharing of knowledge and expertise as well as effective utilisation of personnel. Effective collaboration within nursing education and with other healthcare professionals to achieve higher quality outcomes in an increasingly interdependent higher education system continues to grow in importance
